# Durability of antibody response to vaccination and surrogate neutralization of emerging variants based on SARS-CoV-2 exposure history

**DOI:** 10.1038/s41598-021-96879-3

**Published:** 2021-08-30

**Authors:** Thomas W. McDade, Alexis R. Demonbreun, Amelia Sancilio, Brian Mustanski, Richard T. D’Aquila, Elizabeth M. McNally

**Affiliations:** 1 Northwestern University, 1810 Hinman Avenue, Evanston, IL 60208 USA; 2 Northwestern University Feinberg School of Medicine, Chicago, IL 60611 USA

**Keywords:** Infectious diseases, Viral infection

## Abstract

Two-dose messenger RNA vaccines against the severe acute respiratory syndrome coronavirus 2 (SARS-CoV-2) are highly effective in preventing symptomatic COVID-19 infection. However, the durability of protection is not known, nor is the effectiveness against emerging viral variants. Additionally, vaccine responses may differ based on prior SARS-CoV-2 exposure history. To investigate protection against SARS-CoV-2 variants we measured binding and neutralizing antibody responses following both vaccine doses. We document significant declines in antibody levels three months post-vaccination, and reduced neutralization of emerging variants, highlighting the need to identify correlates of clinical protection to inform the timing of and indications for booster vaccination.

## Introduction

Two-dose messenger RNA vaccines (BNT162b2/Pfizer and mRNA-1273/Moderna) against the severe acute respiratory syndrome coronavirus 2 (SARS-CoV-2) are highly effective in preventing symptomatic SARS-CoV-2 infection^1,2^. However, the durability of protection, particularly with the emergence of viral variants of concern, is not known. Furthermore, the response to vaccination may differ based on prior SARS-CoV-2 exposure history.

## Results

In an IRB-approved community-based serological study, we compared vaccine response in 27 study participants (mean age 39.7 years, 51.9% female, 59.3% Pfizer, 40.7% Moderna). Participants who were seropositive (n = 13) and seronegative (n = 14) prior to vaccination were included. Four seropositive individuals had PCR confirmed COVID-19 infections; remaining seropositive individuals had asymptomatic cases. Samples were collected after the first dose (mean 18.5 days), and twice after the second dose (20.7 days and 56.3 days). Total duration of follow up after first vaccination was approximately 3 months (mean 95.5 days) (Table 1). SARS-CoV-2 receptor binding domain (RBD) IgG was measured with a quantitative immunoassay^3^, and a surrogate virus neutralization test (sVNT) was used to measure inhibition of binding to the ACE2 cell receptor in vitro of wild-type (Wuhan) SARS-CoV-2 spike, as well as the following spike variants: B.1.1.7, B.1.351, and P.1^4^.

**Table 1:** Distribution of study participants, overall and by SARS-CoV-2 exposure history.

	Overall	PCR +	Seropositive	Seronegative
N	27	4	9	14
**Age (years)** 21–30 31–40 41–50 > 50	*N* 10 6 5 6	*N* 0 1 2 1	*N* 7 1 1 0	*N* 3 4 2 5
**Gender** Female Male	*N* 14 13	*N* 3 1	*N* 4 5	*N* 7 7
**Vaccine type** Moderna Pfizer	N 11 16	N 3 1	N 2 7	N 6 8
Timing of blood sampling Dose 1 (days after first dose) Dose 2 (days after second dose) Follow up (days after first dose)	Mean (range) 18.5 (11–23) 19.8 (8–26) 95.5 (70–124)	Mean (range) 14.3 (11–20) 19^a^ 81.0 (70–102)	Mean (range) 19.2 (16–23) 20.0 (8–26) 94.8 (79–124)	Mean (range) 19.3 (17–21) 19.7 (14–26) 100.1 (88–120)

^a^Three PCR + participants were not vaccinated with dose 2.

**Figure 1:**
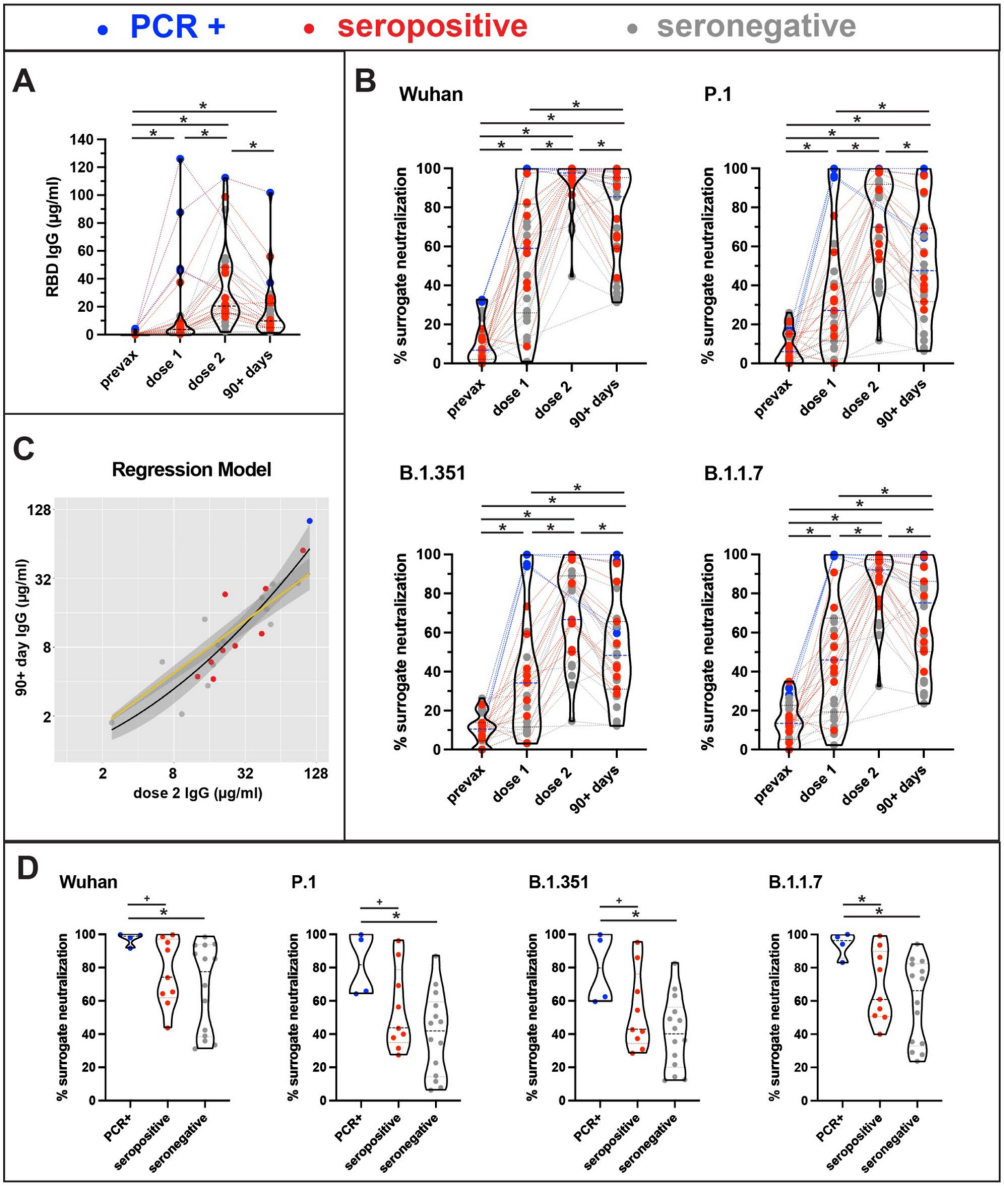
Level of anti-RBD IgG and in vitro neutralization of spike variants following SARS-CoV-2 mRNA vaccination. **(A)** Presents anti-RBD IgG antibody concentration prior to vaccination (prevax), after dose 1 (mean = 18.5 days), after dose 2 (mean = 20.7 days), and 3 months after dose 1 (mean = 95.5 days) for 27 participants. Lines connect results for individual participants, median value is shown with a dotted line, and dot color indicates history of SARS-CoV-2 exposure prior to vaccination (PCR positive confirmed COVID-19, seropositive but asymptomatic, and seronegative). Wilcoxon matched pairs signed-rank tests were used to evaluate statistical significance of median differences. Overall, antibody concentration was significantly lower at 3 months than after dose 2 (p < 0.0001). **(B)** Presents inhibition of spike-ACE2 receptor binding using a surrogate virus neutralization test that measures inhibition of wild-type (Wuhan) spike, as well as the P.1, B.1.351, and B.1.1.7 variants. In comparison with wild-type, neutralization of all variants was significantly lower following dose 1, dose 2, and at 3 months (all comparisons p < 0.0001). Neutralization of each variant was significantly lower at 3 months than after dose 2 (all comparisons p < 0.001). **(C)** Predicts anti-RBD IgG at 3 months as a function of dose 2 response. Regression analysis indicates that the dose 2 response is a strong predictor of the 3 month response (R^2^ = 0.839 (p < 0.0001). A second order in dose 2 showed evidence of a non-linear trend (R^2^ = 0.898; analysis of variance for the nested model p = 0.0023). **(D)** Presents neutralization of each variant at 3 months post-vaccination by SARS-CoV-2 exposure history. Wilcoxon rank-sum test was used to evaluate statistical significance of differences by exposure history. Neutralization of spike-ACE2 interaction did not differ between seropositive and seronegative participants for any variant (all comparisons p > 0.2). Neutralization against all the variants was higher for PCR positive cases in comparison with seropositive and seronegative participants.

Overall, median anti-RBD IgG level increased five-fold after the second vaccine dose in comparison with the first dose (4.2 μg/mL vs 21.0 μg/mL) (Fig. 1A). Median inhibition against wild-type spike was 59.1% after the first dose and 97.7% after the second (Fig. 1B). However, responses were significantly lower to P.1 (27.1% and 70.0%), B.1.351 (34.2% and 66.7%), and B.1.1.7 (45.9% and 92.0%). Median anti-RBD IgG concentration dropped 50.1% at three months post-vaccination relative to the expected peak concentration after the second vaccine dose, and IgG after dose 2 predicted IgG at three months (Fig. 1C). Median inhibition against all the variants was lower at three months, with the largest declines in surrogate neutralization of P.1 (31.2%) and B.1.351 (27.5%), and smaller declines for B.1.1.7 (18.4%) and wild type (12.5%).

We investigated whether pre-vaccination SARS-CoV-2 exposure history influences the magnitude or durability of response to vaccination. Three months after first vaccine dose, median anti-RBD IgG was higher for seropositive participants with a PCR confirmed diagnosis of COVID-19 (27.2 µg/mL) in comparison with participants who were seronegative (8.7 µg/mL), as well as participants who tested seropositive for prior exposure but had asymptomatic infections (8.2 µg/mL). There were no significant differences in levels of surrogate neutralization of spike-ACE2 interaction between pre-vaccination seronegative and seropositive participants (Wilcoxon rank-sum, all comparisons p > 0.70). Neutralization of all the variants was higher for pre-vaccination PCR positive cases in comparison with pre-vaccination seronegative/seropositive participants (Fig. 1D).

## Discussion

We document lower levels of inhibition of spike-ACE2 binding for emerging variants of concern, and significant reductions in anti-RBD IgG and surrogate neutralization of all variants 3 months after a first mRNA vaccine dose. Consistent with a recent report^5^, we find stronger vaccine responses following prior PCR-positive SARS-CoV-2 infection. Importantly, these stronger responses were limited to participants with PCR confirmed cases of COVID-19, and were not seen among those who did not experience symptoms or were seronegative. A large proportion of SARS-CoV-2 infections are asymptomatic^6^, and our results indicate that seropositivity alone is not sufficient to predict a robust antibody response to vaccination. Recent data suggest that lower responses to vaccination are associated with increased risk of breakthrough infection: Among vaccinated healthcare workers in Israel monitored from January to April 2021, neutralizing antibody and anti-spike IgG titers were significantly lower in workers with breakthrough infections in comparison with matched, uninfected controls^7^. The B.1.1.7 variant was detected in 85% of the breakthrough infections. While antibody neutralization of emerging variants may be reduced in comparison with the ancestral strain of SARS-CoV-2, T cell reactivity following vaccination or natural infection has been shown to be similar across strains and may reduce the severity of COVID-19 if a breakthrough infection occurs^8^. The decline in antibody levels over three months post-vaccination, and the relatively reduced neutralization of variants of concern, point to an urgent need to identify correlates of clinical protection to inform the timing of and indications for booster vaccination.

## Methods

### Study design

A subset of participants that were enrolled in a larger seroprevalence study of over 8000 residents of the Chicagoland area (Screening for Coronavirus Antibodies in Neighborhoods (SCAN)), provided multiple, self-collected finger stick dried blood spot (DBS) samples from April 2020 through May 2021^9^. Participants provided consent online and completed a survey regarding COVID-19 status and symptoms. Participants received materials for finger-stick DBS collection through the mail or on-site. DBS samples were returned through the mail or on-site. In January and February 2021, participants completed an additional survey reporting COVID-19 vaccination status and dates of vaccination and were asked to provide a DBS sample approximately three weeks after receiving dose 1, two weeks after receiving dose 2, and then again approximately 60 days after receiving dose 2. Participants were categorized based on pre-vaccination RBD IgG serology. Participants were categorized as COVID-19 PCR + if they reported testing positive for SARS-CoV-2 on a clinical molecular diagnostic test for acute infection any time prior to vaccination. Participants who lacked self-report of a positive SARS-CoV-2 clinical diagnostic test result were categorized as seropositive if anti-RBD IgG antibodies were detected in DBS samples collected between April and December 2020, while those without detectable levels of RBD IgG were categorized as seronegative. All research activities were performed in accordance with relevant guidelines and regulations with protocols approved by the institutional review board at Northwestern University (#STU00212457 and #STU00212472). All participants provided informed consent.

### Anti-RBD IgG assay

Antibodies against SARS-CoV-2 receptor binding domain (RBD) were quantified with an established protocol validated for use with DBS samples which shows high agreement with results from matched serum samples (R = 0.99)^3,10^. Results were normalized to the CR3022 antibody with known affinity to RBD^11^. Anti-RBD IgG concentration (µg/ml) was calculated from the four parameter logistic regression of the CR3022 calibration curve. A value > 0.39 µg/ml CR3022 was considered seropositive.

### Surrogate virus neutralizing test

A multiplex competitive immunoassay was used to quantify neutralizing activity (% inhibition) of spike-ACE2 interaction in vitro. The protocol was previously validated for DBS, with high agreement with results from matched serum samples (R = 0.99)^4^. Samples were incubated with human recombinant ACE2 conjugated with an electrochemiluminescent label in assay wells coated with an array of SARS-CoV-2 spike protein variants, including wild type, D B.1.1.7, B.1.351, P.1, and D614G (Meso Scale Discovery K15436U). The Meso Scale Discovery QuickPlex SQ 120 Imager was used to read mean fluorescence intensity (MFI), and % inhibition was calculated as follows: % inhibition = 100 × 1 − (sample MFI/negative control MFI). Prior validation studies indicate that results from the surrogate virus neutralization method correlate highly with results from conventional live virus (Pearson R = 0.93) and pseudovirus neutralization assays (R = 0.92)^12^.

## References

[1] Polack FP (2020). Safety and efficacy of the BNT162b2 mRNA Covid-19 vaccine. N. Engl. J. Med..

[2] Baden LR (2021). Efficacy and safety of the mRNA-1273 SARS-CoV-2 vaccine. N. Engl. J. Med..

[3] McDade T (2020). High seroprevalence for SARS-CoV-2 among household members of essential workers detected using a dried blood spot assay. PLoS ONE.

[4] Sancilio AE (2021). A surrogate virus neutralization test to quantify antibody-mediated inhibition of SARS-CoV-2 in finger stick dried blood spot samples. Sci. Rep..

[5] Saadat S (2021). Binding and neutralization antibody titers after a single vaccine dose in health care workers previously infected qith SARS-CoV-2. JAMA.

[6] Oran DP, Topol EJ (2020). Prevalence of asymptomatic SARS-CoV-2 infection: A narrative review. Ann. Intern. Med..

[7] Bergwerk M (2021). Covid-19 breakthrough infections in vaccinated health care workers. N. Engl. J. Med..

[8] Tarke A (2021). Impact of SARS-CoV-2 variants on the total CD4+ and CD8+ T cell reactivity in infected or vaccinated individuals. Cell Rep. Med..

[9] Demonbreun AR (2021). Patterns and persistence of SARS-CoV-2 IgG antibodies in a US metropolitan site. J. Clin. Investig..

[10] Amanat F (2020). A serological assay to detect SARS-CoV-2 seroconversion in humans. Nat. Med..

[11] Yuan M (2020). A highly conserved cryptic epitope in the receptor binding domains of SARS-CoV-2 and SARS-CoV. Science.

[12] Tan CW (2020). A SARS-CoV-2 surrogate virus neutralization test based on antibody-mediated blockage of ACE2–spike protein–protein interaction. Nat. Biotechnol..

